# Prehabilitation: tertiary prevention matters

**DOI:** 10.1093/bjs/znae028

**Published:** 2024-03-04

**Authors:** Heleen Driessens, Allard G Wijma, Carlijn I Buis, Maarten W Nijkamp, Gertrude J Nieuwenhuijs-Moeke, Joost M Klaase

**Affiliations:** Department of Hepato-Pancreato-Biliary Surgery and Liver Transplantation, University Medical Centre Groningen, Groningen, The Netherlands; Department of Hepato-Pancreato-Biliary Surgery and Liver Transplantation, University Medical Centre Groningen, Groningen, The Netherlands; Department of Hepato-Pancreato-Biliary Surgery and Liver Transplantation, University Medical Centre Groningen, Groningen, The Netherlands; Department of Hepato-Pancreato-Biliary Surgery and Liver Transplantation, University Medical Centre Groningen, Groningen, The Netherlands; Department of Anaesthesiology, University Medical Centre Groningen, Groningen, The Netherlands; Department of Hepato-Pancreato-Biliary Surgery and Liver Transplantation, University Medical Centre Groningen, Groningen, The Netherlands

## Introduction

Prehabilitation is linked to the concept of prevention. It is, however, distinct from primary prevention, which focuses on averting disease onset, and secondary prevention, which involves early detection of a disease. Prehabilitation falls within the domain of tertiary prevention, specifically targeting the prevention of treatment-related complications and the mitigation of their impact. This becomes crucial for patients who must deal with their disease and need surgery, as primary and secondary prevention come too late. The importance of tertiary prevention for these patients cannot be overstated. There are three key reasons underlying the significance of tertiary prevention.

First, complications directly diminish a patient’s quality of life both in the short-term and the long-term, sometimes leading to dependency or even death^1^. In cancer surgery, complications can detrimentally affect cancer-specific survival rates^2,3^. Second, complications contribute to a significant rise in hospital admission costs. Up to 30% of the budget allocated to colorectal surgery is consumed by complications^4^. Redirecting this funding towards prehabilitation interventions at the outset could substantially enhance surgical outcomes. Notably, a correlation exists between total hospital admission costs and the comprehensive complication index^5^. This emphasizes that not only the most severe but every additional complication has impact. Third, the incidence of complications after major surgery is structurally high. Major abdominal surgery is associated with up to 30% major complications (Clavien–Dindo grade greater than or equal to IIIA), up to 30% minor complications, and up to 25% unplanned readmissions^6,7^. Currently, approximately one in three patients undergoing major abdominal surgery is aged 75 years or older. Elderly patients, often burdened with multiple co-morbidities, face an elevated risk of complications, particularly cardiopulmonary issues that can lead to prolonged hospitalization^8^. As the population continues to age, the surgical community is confronted with an increasingly important task: addressing the challenges of tertiary prevention.

The outcome of surgical care is influenced by multiple factors. Notably, hospitals have made strides in enhancing their structures and processes. The introduction of Enhanced Recovery After Surgery (ERAS) programmes marked a significant advancement, including incorporation of widespread use of minimally invasive surgery. This resulted in decreased complication rates and improved the speed of functional recovery^9^. Additionally, centralizing highly complex, low-volume surgical procedures led to a noteworthy reduction in failure-to-rescue and mortality rates^10,11^. Mandatory audits further bolstered surgical outcomes^12,13^. However, amidst these advancements, limited attention has been directed towards patients themselves. Particularly, minimal consideration has been given to patient-related modifiable risk factors that could be optimized during the pre-surgery waiting interval. Most risk factors, which serve as case-mix correction elements in mandatory audits for comparing hospital performance, remain unmodifiable. Examples include age, sex, tumour stage, co-morbidity, and the timing of the operation (elective or (sub)acute). On the other hand, cardiopulmonary or aerobic fitness stands out as a critical modifiable risk factor, next to other modifiable risk factors, such as nutritional status, psychological resilience, anaemia, regulation of co-morbidities, frailty, and intoxications. In this review, the authors aim to: summarize the current evidence; summarize their own prehabilitation experience; and point out recommendations for future directions.

## Review of the current evidence

### Prehabilitation across different surgical specialties

Prehabilitation is mainly studied in cancer surgery. However, prehabilitation programmes are applied across a wide variety of surgical specialties, including abdominal, cardiovascular, orthopaedic, and gynaecological surgery. Within cancer surgery, prehabilitation programmes have been most frequently studied within the context of colorectal surgery.

A systematic review on major abdominal cancer surgery showed that prehabilitation can lead to a reduction in postoperative complications, but was not associated with shorter length of hospital stay, intensive care unit admission, and mortality^14^. Heterogeneity in the applied interventions, outcome measure used, and patient category involved makes comparison of studies difficult. A more recent meta-analysis on prehabilitation before major abdominal cancer surgery did show a reduction in length of stay, but no reduction in complications^15^. In cardiovascular surgery, prehabilitation programmes are associated with a reduced complication rate and shorter length of hospital stay, but, again, considerable heterogeneity in the protocols and insufficient sample sizes hamper accurate meta-analysis^16^. Therefore, the effectiveness of prehabilitation in cardiovascular surgery is still to be determined. For patients undergoing orthopaedic surgery, prehabilitation may lead to improved functioning after total knee and hip replacement and lumbar surgery^17^. Studies on prehabilitation in gynaecological oncology surgery are scarce. The results of some of these studies showed a decrease in length of hospital stay and shorter time to adjuvant chemotherapy, but other studies did not show these improvements^18^.

The differences in the results on prehabilitation across different surgical specialties highlight the difficulty in determining the true effectiveness of prehabilitation across different patient populations.

### Prehabilitation during neoadjuvant treatment

A major challenge is the increased use of neoadjuvant therapy in surgical oncology, with concomitant physical decline^19^. The start of prehabilitation should be transferred to an earlier point in a patient’s trajectory, instead of commencing it in the weeks before surgery. The goal of prehabilitation in these circumstances is not to improve fitness but rather to maintain fitness or to minimize its decline due to neoadjuvant therapy. It has been shown in several studies that prehabilitation during neoadjuvant chemotherapy is feasible and probably effective^20,21^. An even better tumour response has been observed in advanced rectal and oesophageal cancer^22,23^. Logistics and a broad collaboration between different care providers are essential to achieve a workable programme.

### Types of prehabilitation programmes

Initially, the main focus of prehabilitation programmes was exercise-based prehabilitation. Most of these programmes were unimodal, addressing only aerobic fitness^24^. Prehabilitation as a multimodal intervention to withstand the surgical stress of an operation is, however, more than improving cardiorespiratory fitness and muscle function alone. It also involves looking at other patient-related modifiable risk factors. Over time, the focus has shifted towards multimodal programmes, allowing for a more holistic patient approach^24^. These programmes often consist of a combination of interventions targeting modifiable risk factors, including two or more of the following: an exercise programme; a dietary intervention focused on improving nutritional status; a psychological programme improving mental resilience; treatment of anaemia; optimization of poorly regulated co-morbidities, such as diabetes; screening for frailty; and guidance on quitting smoking and alcohol. Of these modifiable risk factors, the main focus is still aerobic fitness, as this is considered the most important risk factor for postoperative outcomes^25^.

### Aerobic fitness

Aerobic fitness encompasses the body’s ability to absorb oxygen (pulmonary function), transport oxygen (cardiovascular system function), and utilize oxygen within the muscle’s mitochondrial system (muscle function). Aerobic fitness can be assessed objectively using a cardiopulmonary exercise test (CPET)^26^. From the CPET results, both VO_2_ peak (maximum rate of oxygen consumption attainable during maximal effort) and VO_2_ at the ventilatory anaerobic threshold (VAT) (a valuable submaximal indicator when maximal effort cannot be reached) can be derived. Extensive research within the surgical domain has explored the correlation between the VAT and the risk of complications. In general, patients exhibiting a VAT less than 11 ml/kg/min face an elevated risk of complications after various types of major abdominal surgery^27,28^. This knowledge holds promise for risk stratification, particularly in elderly patients undergoing major abdominal or thoracic surgery. Patients aged 75 years or older, who are also physically unfit, exhibit an increased risk of postoperative mortality after liver resection^29^. Moreover, those with a VAT less than 11 ml/kg/min experience prolonged ICU stays after liver transplantation and an increased overall mortality rate^30^. A CPET does require specific equipment, expertise, and time investment, making it a costly aerobic fitness test. Less sophisticated exercise tests (for example the (modified) steep ramp test (SRT) and the incremental shuttle walk test) could serve as practical alternatives to estimate aerobic capacity^31^. Low preoperative aerobic fitness, assessed using a SRT, has been associated with the development of postoperative complications^31^. The SRT is also strongly correlated with VO_2_ peak, assessed using a CPET, and the SRT has a much shorter duration than the CPET, making it a brief and reliable alternative^32^.

As aerobic fitness is by far the best modifiable risk factor and can be improved by a prehabilitation programme, this risk factor should be assessed routinely in preoperative patient care^25^. By improving cardiorespiratory function and muscle strength before an operation, patients start at a higher fitness level. After surgery, there is a physiological decline in physical functioning, but patients will not reach the critical zone, which may lead to complications, dependency, and even death. In contrast, without prehabilitation, patients generally experience a loss of functional capacity in the waiting time before surgery. The corresponding author’s group combined risk stratification and prehabilitation^33^. Patients at high risk of complications because of low cardiopulmonary fitness (defined as a VAT less than 11 ml/kg/min), who were scheduled for colorectal resection, were randomized to either enter a prehabilitation programme (consisting of high-intensity interval training (HIIT) three times a week for 3 weeks combined with muscle-strength training supervised by a community physical therapist) or standard care. In this trial in 57 patients, prehabilitation decreased the number of patients with complications from 72.4% to 42.9% (relative risk 0.59, 95% c.i. 0.37 to 0.96; *P* = 0.024). The VAT increased by a median of 10.1% in the group who underwent an exercise programme. The recently published international randomized PREHAB trial in 251 patients with colorectal cancer demonstrated the benefit of a 4-week in-hospital supervised multimodal prehabilitation programme, with a reduction in severe complication rate (comprehensive complication index (CCI) score of greater than or equal to 21) (17.1% *versus* 29.7%; OR 0.47, 95% c.i. 0.26 to 0.87; *P* = 0.02)^34^. Fewer medical complications were encountered compared with patients receiving standard care (15.4% *versus* 27.3%; OR 0.48, 95% c.i. 0.26 to 0.89; *P* = 0.02). In this trial, prehabilitation was offered to all patients and not only to a selection of high(er)-risk patients. An emulated target trial in 251 elderly colorectal cancer patients (aged 65 years or older and/or having an ASA grade greater than or equal to III) showed improved postoperative outcomes after a multimodal prehabilitation programme of 3 weeks^35^. In the intention-to-treat analysis, prehabilitation reduced the risk of complications from 0.69 to 0.45. Patients who underwent prehabilitation had a 55% lower CCI score and a shorter hospital stay (7.3 days *versus* 4.9 days). Importantly, all three of the studies mentioned above for patients with colorectal cancer showed the effect of tertiary prevention by prehabilitation, expressed as a reduction in complications and their severity, at least but not alone in high-risk populations. A HIIT programme of 3–4 weeks seems sufficient to improve aerobic fitness and should be an integral part of an exercise prehabilitation programme before major surgery according to a recent meta-analysis^36^.

Given the correlation between a higher level of objectively measured physical activity during the interval preceding surgery and faster postoperative functional recovery^37^, an intriguing question emerges. Is it possible to increase physical activity in a simple way before major surgery? A recent review article underscored the feasibility of enhancing preoperative physical activity^38^. Interestingly, outcomes based on self-reported physical activity measures tend to yield more substantial effects when compared with objectively measured outcomes. To further advance the field, a stronger emphasis on high-quality research using objective measurements is warranted. In this context, the utilization of wearables, including activity trackers, during the perioperative phase holds great promise. Currently, it is possible to monitor the impact of and recovery after pancreatic cancer treatment, including (neo)adjuvant therapy and surgery, using data from devices that can be worn by patients, for example smartwatches^39^.

## Experience of the authors’ own group

### Multimodal prehabilitation programme

**Fig. 1:**
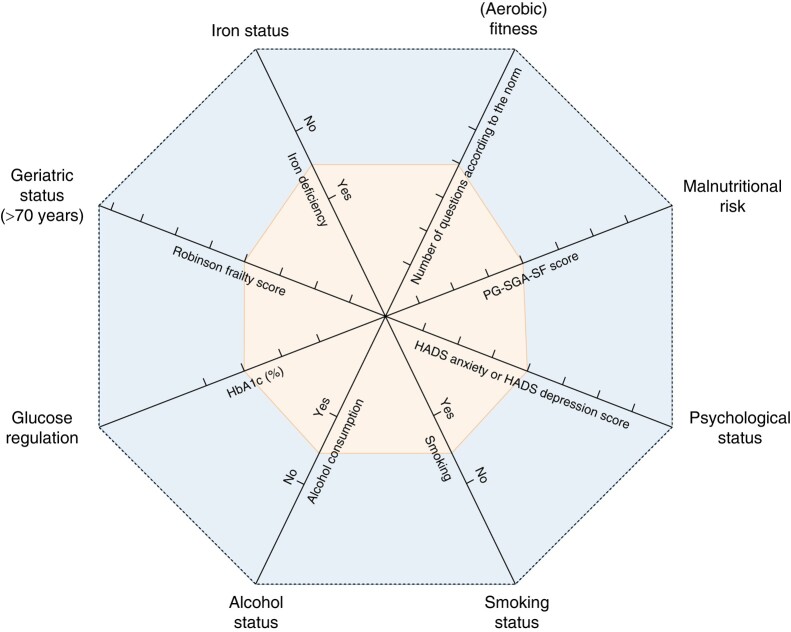
Model of eight patient-related (partly) modifiable risk factors PG-SGA-SF, Patient-Generated Subjective Global Assessment Short-Form; HADS, Hospital Anxiety and Depression Scale.

At the authors’ prehabilitation outpatient clinic they have designed a model consisting of eight (partly) modifiable risk factors, including aerobic (cardiopulmonary) fitness, nutritional status, psychological resilience, smoking status, alcohol status, glucose regulation, geriatric status, and iron status (haemoglobin level) (*Fig. 1*, made available by and in courtasy of Marcel den Dulk, Maastricht University Medical Centre). According to the framework for prehabilitation services, the process of prehabilitation includes screening, assessment, intervention, and reassessment^40^. Interventions are delivered together, have complementary benefit, and should be considered as a care bundle. The authors reported that two-thirds of the first 100 patients scheduled for hepatic, pancreatic, and biliary surgery needed at least one or two interventions^41^.

- For aerobic fitness, the authors screen patients using four questions adapted according to the clinical guideline and recommendations on preoperative exercise training in patients awaiting major non-cardiac surgery^42^: is the patient aged above 70 years old?; does the patient suffer from a poorly controlled chronic health condition (co-morbidity)?; does the patient need neoadjuvant therapy?; and does the patient ‘not’ meet the Dutch Physical Activity Guideline (physical activity of moderate intensity for at least 150 min every week and muscle-strength and balance exercises twice a week)? If the answer to any of the questions is yes, the authors send the patient for objective assessment of cardiopulmonary fitness, assessed using a CPET. When the VAT is less than 11 ml/kg/min, the authors counsel the physically frail patient for a supervised home-based HIIT programme. All other patients receive personalized exercise instruction from the sports medicine department. For less physically frail patients, an exercise prehabilitation programme can also be offered at a gym under the supervision of a personal trainer. After 4 weeks, the authors perform a reassessment using a CPET for the patients who follow the home-based programme. Physically frail patients who do not respond to exercise prehabilitation are very prone to complications and surgery should at least be reconsidered in a multidisciplinary team meeting.
- For screening of nutritional status, the authors use the Patient-Generated Subjective Global Assessment Short-Form (PG-SGA-SF) questionnaire^43^. Patients with a score of greater than or equal to 4 are assessed by a dietician. The intervention comprises a minimum amount of 1.5 g protein/kg bodyweight/day and an extra amount of 30 g whey protein after every training session and before sleep, vitamins, and pancreatic enzyme replacement therapy (PERT) in the case of pancreatic surgery, and patients are asked to keep a food diary. Reassessment involves using the PG-SGA-SF questionnaire, which can be used as a monitoring instrument, and using bodyweight measurement.
- Psychological resilience is screened using the Hospital Anxiety and Depression Scale (HADS) score. When the HADS score is greater than or equal to 8, a personalized psychological intervention can be provided. Thorough information and explanation regarding a patient’s near-future trajectory and proper support provided by a case manager, taking into account coping behaviour and health literacy, are important^44^.
- Support in quitting smoking is provided and patients are advised to contact their general practitioner for guidance on quitting smoking.
- Support in quitting alcohol is provided.
- As hyperglycaemia is related to wound complications and anastomotic leakage, the authors recently added HbA1c as a screening measure^45,46^. A next step will be continuous perioperative glucose monitoring in patients with all types of diabetes.
- A frailty screening test like the Robinson Frailty Score can be used to select patients for a comprehensive geriatric assessment by a geriatrician. There is a relationship between this frailty score and complications after colorectal surgery^47^. The geriatrician plays a role in, amongst others, preventing delirium and addressing polypharmacy and multimorbidity.
- As anaemia is a risk factor for complications like anastomotic leakage, iron infusion to correct iron deficiency anaemia could diminish complications and reduces the number of red blood cell transfusions^46,48^. It is also important to correct iron deficiency without anaemia, as iron deficiency leads to the reduced availability of myoglobin and the dysfunction of mitochondria in skeletal muscle, with myoglobin and correctly functioning mitochondria both being essential for exercise prehabilitation^49^.

Recently, the authors conducted a 1 : 1 propensity score matched study in 120 patients who participated in their prehabilitation programme before pancreatic surgery. The authors found a clinically relevant reduction in cardiopulmonary complications (from 23.3% to 9.2%; *P* = 0.002) for the patients who underwent prehabilitation^50^. Therefore, the authors’ experience is that this prehabilitation programme is able to reduce postoperative complications and is of benefit to their patients.

What the corresponding authors’ group learned from the first randomized study on prehabilitation was that trial accrual was very slow^33^. Patients who are not fit are reluctant to start training, because of transport and financial constraints, limited time, and physical ailments. Moreover, the psychological aspect of having cancer played a role in the patients’ perception that the operation had to be performed as soon as possible, with nearly no waiting time. Together with a thorough explanation that waiting time would not be prolonged, but instead used as preparation time, not affecting cancer prognosis^51^, the authors developed a 4-week semi-supervised home-based prehabilitation programme. A specialized ergometer for HIIT was brought to a patient’s home, where the patient did their exercise training under the supervision of a physical therapist. This training programme was highly successful in unfit patients scheduled for liver and pancreatic surgery, with a mean increase in the VAT of 17.8%. The authors believe that the reported high improvement in aerobic fitness with an adherence rate of 83% is due to the combination of the semi-supervised way of providing exercise training and delivery at home^52^. Insights into the perspectives of patients enabled the authors to better inform patients on prehabilitation and offer a semi-supervised home-based prehabilitation programme, thereby increasing the rate of compliance.

### Implementation of prehabilitation

Incorporating prehabilitation into standard preoperative care necessitates restructuring of the preoperative care pathway, mirroring the methodology adopted within the authors’ outpatient prehabilitation clinic^41^. The incorporation of prehabilitation into standard care is heavily reliant on efficient logistical organization. The blueprint developed by the authors, centred around eight (partly) modifiable risk factors, stands as an exemplary working framework that other medical institutions can emulate (*Fig. 1*).

Undoubtedly, prehabilitation interventions should ideally be made available in proximity to a patient’s residence whenever feasible. This approach will enhance patient compliance, while simultaneously curbing expenses. Presently, the orchestration of screening, assessment, prehabilitation intervention planning, and subsequent reassessment falls under the purview of the hospital. Nevertheless, considering the prospective evolution of prehabilitation into a prevailing practice, it is conceivable that the future prehabilitation process will commence with the involvement of the general practitioner.

## Future directions of prehabilitation

### Homogeneity in interventions, target patient groups, and outcome measures

The evidence concerning prehabilitation is rising. However, according to recent reviews, it is currently not robust enough for prehabilitation to be recommended in clinical guidelines^14,53^. This is caused by heterogeneity in interventions, target patient groups, and outcome measures. The top three research priorities in prehabilitation listed in an international Delphi study were: the effects of prehabilitation on surgical outcomes; identifying populations most likely to benefit from prehabilitation; and the optimal composition of prehabilitation programmes^54^. Concerning the first priority, a core outcome set could be helpful^55^. Regarding the second priority, a clear definition of high-risk patients is imperative. Age itself is not distinctive enough. Cardiopulmonary fitness level, an important target for prehabilitation, should most likely be added. As body composition derived from segmentation of a CT slide at the L3 level has been consistently related to surgical outcome, with low muscle mass, myosteatosis (deprived muscle function), and high visceral fat mass all being risk factors for postoperative morbidity, it is time to also incorporate body composition into standard risk stratification before major surgery^56,57^. If a prehabilitation programme lasts longer than the 3–4 weeks before elective major surgery, for example for patients on the waiting list for liver transplantation, it could positively influence body composition, as it has been shown to increase muscle mass and to lower fat mass^58^.

A uniform design for the content of a prehabilitation programme and a core set of outcome measures might be helpful for future research, enabling outcomes of prehabilitation for different populations of surgical patients to be compared. This might aid in the determination of which patient population might benefit most from prehabilitation.

### Financial aspects of prehabilitation

Broad implementation of prehabilitation is hampered by the lack of reimbursement by insurance companies that do recognize prehabilitation as very promising, but consider scientific evidence too fragile. Therefore, hospital-board directors are reluctant, in general, to invest in a prehabilitation service, although cost-effectiveness is possible, considering the high costs resulting from complications. Barberan-Garcia *et al*.^59^ showed the financial benefit of prehabilitation in a cost-consequence analysis of their randomized prehabilitation trial of high-risk patients awaiting major surgery. The authors’ group demonstrated with a theoretical financial algorithmic that the return on investment for prehabilitation in pancreatic surgery was 1.55^60^. In addition, the Working Group on Prehabilitation of the Dutch Society of Surgery and the Knowledge Institute of the Federation of Medical Specialists recently conducted a budget impact analysis for patients undergoing colorectal surgery in the Netherlands^61^. In a case-based analysis, they showed that the implementation of a multimodal prehabilitation programme is expected to result in healthcare cost savings of approximately €12.8 million per year. Over a 5-year interval, this could potentially lead to cost savings of around €64.3 million in such a small country with almost 18 million inhabitants.

### Mechanisms underlying prehabilitation—the unknowns

The effects of prehabilitation on the underlying mechanistic factors that contribute to reduced tolerance to surgical stress and the development of postoperative complications, such as patients’ neuroendocrine, metabolic, and immune status and dynamics, remain largely unknown or, at best, theoretical^62^. Despite substantial growth in prehabilitation research, only a few attempts have been made to delve into these fundamental mechanisms. To enable the effective transition of prehabilitation into an insured care package, accessible to all patients who will potentially benefit, it becomes imperative to gain a deeper understanding of the systemic and molecular factors that underlie the success and failure of prehabilitation, including a better insight into the development of postoperative complications. This understanding will help to identify patients who stand to gain from prehabilitation beforehand and enable the development of a patient-tailored personalized approach, moving away from a one-size-fits-all prophylactic approach.

### Need for more evidence

Up to now, most evidence has been collected from patients with colorectal cancer; more evidence is required from multicentre studies conducted by hospitals that already offer prehabilitation. For patients undergoing pancreatic surgery, a nationwide stepped wedge cluster randomized trial for prehabilitation implementation (ClinicalTrials.gov ID NCT05851534) has been initiated. As prehabilitation has been extensively reported to be safe, even among high-risk populations, such as patients awaiting solid organ transplants^63^, and has consistently demonstrated non- inferiority to standard care in randomized trials, it raises questions about the continued necessity of relying solely on such trials. By embracing prehabilitation as standard practice, the opportunity arises to pioneer a holistic approach to fostering healthier lifestyles across all hospital care pathways—encompassing both pretreatment and post-hospitalization phases. Over time, convergence of tertiary prevention and primary prevention becomes increasingly plausible. The ‘teachable moment’ of facing a cancer diagnosis or major surgery often serves as a transformative moment that likely prompts many patients to adopt healthier lifestyle behaviours beyond their hospitalization interval. Nonetheless, actual real-life data on this subject remain limited. To ensure a cohesive link between prehabilitation and rehabilitation, the integration of prehabilitation must be visibly woven into hospital practices, thereby solidifying prehabilitation as an integral component of ERAS programmes^64^.

## Conclusion

Prehabilitation is unquestionably positioned to achieve the Quadruple Aim^65^: enhancing the quality of care through tertiary prevention; curbing costs; incorporating and improving patients’ experience; and fostering satisfaction among healthcare professionals engaged in delivering prehabilitation programmes. Patients stand to gain significantly, as they receive patient-centred care that offers guidance throughout an otherwise uncertain preoperative waiting interval, ultimately transforming it into a valuable phase of effective preparation under their own control. Globally, a staggering 310 million individuals undergo major surgeries annually, a figure projected to rise, owing to an expanding and ageing population, alongside enhanced healthcare access^66^. As stated by Wynter-Blyth and Moorthy^67^ in their editorial, in essence, from a statistical standpoint, everyone is in a preoperative state. The moment has arrived to integrate prehabilitation into everyday medical practice. The full realization of this integration will only be achieved when patients actively request their prehabilitation programme rather than solely inquiring about their surgery date.

## Data Availability

Not applicable.
